# Reappraising Use of Flecainide for Atrial Fibrillation and Ventricular Arrhythmias in Structural Heart Disease Patients

**DOI:** 10.3390/medicina61101845

**Published:** 2025-10-15

**Authors:** Dimitrios Tsiachris, Sotirios C. Kotoulas, Ioannis Doundoulakis, Christos-Konstantinos Antoniou, Michail Botis, Konstantinos Pamporis, Nikolaos Argyriou, Aikaterini-Eleftheria Karanikola, Panagiotis Tsioufis, Athanasios Kordalis, Konstantinos Tsioufis

**Affiliations:** First Department of Cardiology,“Hippokration” General Hospital, National and Kapodistrian University, Vasilissis Sofias 112, 11527 Athens, Greece; soter96@icloud.com (S.C.K.); doudougiannis@gmail.com (I.D.); ckantoniou@hotmail.gr (C.-K.A.); mgmpotis94@gmail.com (M.B.); konstantinospab@gmail.com (K.P.); nikos_ar@hotmail.com (N.A.); elinakaranikola@gmail.com (A.-E.K.); ptsioufis@gmail.com (P.T.); akordalis@gmail.com (A.K.); ktsioufis@gmail.com (K.T.)

**Keywords:** Ic antiarrhythmics, flecainide, structural heart disease, coronary heart disease, ventricular arrhythmias, atrial fibrillation

## Abstract

*Background*: Flecainide, a class Ic antiarrhythmic agent, has long been contraindicated in structural heart disease (SHD) due to findings of the Cardiac Arrhythmia Suppression Trial (CAST). However, its proven efficacy in patients without structural abnormalities and emerging safety data in selected SHD populations have prompted reconsideration of its role. *Aim*: This mini review evaluates recent evidence on the safety and efficacy of flecainide in atrial fibrillation (AF) and premature ventricular contractions (PVCs), particularly in patients with stable coronary artery disease (CAD), and arrhythmogenic right ventricular cardiomyopathy (ARVC). *Results*: Modern imaging and improved risk stratification allow for more precise identification of patients who may safely receive flecainide, even in the presence of specific structural abnormalities. Observational studies have reported no mortality or ventricular arrhythmias incidence increase in stable CAD or ARVC when flecainide is administered under stringent criteria. While current guidelines remain cautious, clinical practice is beginning to reflect a more individualized approach. *Conclusions*: Flecainide use in selected SHD patients appears both feasible and safe when guided by comprehensive imaging and clinical judgment. The need for prospective randomized trials to confirm these findings and potentially inform future guideline updates is urgent and of utmost importance in the field of antiarrhythmic therapies.

## 1. Introduction

Flecainide is a class Ic antiarrhythmic agent with potent sodium channel-blocking properties and well-established efficacy in treating atrial fibrillation (AF) and premature ventricular contractions (PVCs) in structurally normal hearts [1,2]. However, its use in patients with structural heart disease (SHD) has been restricted, following the Cardiac Arrhythmia Suppression Trial (CAST), which showed increased mortality in post-myocardial infarction (MI) patients with reduced left ventricular ejection fraction receiving class Ic agents [3].

This legacy has led to guideline-based contraindications that broadly categorize many forms of SH, Dincluding stable coronary artery disease (CAD), left ventricular hypertrophy and mild valvular disease, as high-risk settings for flecainide use [1,2]. Yet, recent observational studies and critical reviews have questioned this sweeping approach [4,5]. Experts have pointed out that the CAST enrolled a very specific high-risk post-MI population with a significant scar burden and active ischemia, which may not be representative of contemporary patients with extensive revascularization and stable ischemic or nonischemic SHD [4,5].

The core concern limiting use of flecainide in SHD is the fear of ventricular proarrhythmia, primarily based on the CAST findings. However, newer retrospective and real-world data challenge the continued relevance of this concern in contemporary practice [6,7,8,9]. Several cohort studies have now reported favorable outcomes with flecainide in patients with Arrhythmogenic Right Ventricular Cardiomyopathy (ARVC) [7], stable CAD [6], and other nonischemic cardiomyopathies [3]. While current guidelines have not yet changed, these findings support the need for prospective randomized trials. Studies like FLECA-ED [8,9] demonstrate the feasibility and safety of structured flecainide use in well-characterized cohorts, providing a framework for future SHD-focused research. The aim of our study is to present all relevant data of flecainide use in patients with different types of SHD.

The aim of this mini review is to compile all the relevant data on flecainide use in patients with different types of SHD. A comprehensive and structured search of PubMed/MEDLINE, Embase and Cochrane Library databases was conducted, covering the period from 1991, year of the CAST, to May 2025. The search utilized a range of key terms, including “flecainide”, “structural heart disease”, “atrial fibrillation”, “arrhythmogenic right ventricular cardiomyopathy”,” ventricular arrhythmia”, “premature ventricular contractions”, “PVC-induced cardiomyopathy”,” left ventricular hypertrophy”.

### 1.1. Old and New Evidence

#### 1.1.1. CAST: Overgeneralization to Structural Heart Disease

Recommendations to avoid the use of class 1c agents in patients with AF and SHD have been influenced primarily by exclusion of these patients from trials after the CAST results, rather than driven by observed adverse effects in large-scale clinical studies [1,2]. CAST assessed the impact of antiarrhythmic therapy (including flecainide) in patients with previous MI, left ventricular dysfunction, and PVCs in an era of poor revascularization [3,10]. At that time, amiodarone was considered too toxic to be included [10]. Unexpectedly, antiarrhythmic drug treatment that had been intended to suppress ventricular arrhythmias significantly increased arrhythmic and all-cause mortality attributed to residual ischemia and myocardial scar [3,10]. Consequently, flecainide was contraindicated in patients with CAD and ischemic cardiomyopathy. Practice guidelines extended contraindications to all class Ic agents, including propafenone and all other forms of SHD, including left ventricular hypertrophy and cardiomyopathy, despite the absence of relevant data [1,2].

More specifically, the CAST population included patients with recent MI since most (8/10) were enrolled in the acute post MI phase, as well as poor revascularization, with percutaneous coronary intervention performed in 19% and coronary artery bypass graft in another 19% [3,10]. CAST sub-analysis revealed that it was the presence of non-Q wave MI that was related to sudden cardiac death events and all-cause mortality in patients receiving encainide or flecainide [3,10,11,12]. It has been shown that conduction slowing in an active ischemic substrate facilitates malignant ventricular arrhythmias [13]. These facts signify the dominant role of residual or active ischemia as a basic modulator of flecainide proarrhythmia [10,11,12,13].

#### 1.1.2. Flecainide in Patients with AF and CAD

Flecainide constitutes one of the most effective rhythm control agents for patients with paroxysmal AF and structurally normal hearts [1]. It offers rapid onset, high arrhythmia suppression rates, and excellent tolerability when prescribed under appropriate safety protocols, such as baseline echocardiography and exercise testing to exclude ischemia or latent conduction disease [1].

In the setting of AF cardioversion in emergency department, we previously conducted a network meta-analysis that identified vernakalant and flecainide as the most effective, as well as safe antiarrhythmics for pharmacologic cardioversion over different time settings [14]. In this framework, the effectiveness and safety of class Ic agents for cardioversion of paroxysmal AF, in patients with and without SHD [15] was specifically addressed. Based on these data, intravenous flecainide appeared to be more effective compared to propafenone since only 2 patients need to be treated in order to cardiovert one. The breakthrough finding of this analysis is practically the absence of ventricular proarrhythmia in the whole study population (>1500 patients, with 365 of them suffering from SHD, basically CAD) taking into account that only 2 cases with ventricular tachycardia were recorded based on studies conducted in the early 1990s [16,17]. Surprisingly, no life-threatening ventricular arrhythmias or death were reported in the outdated studies that assessed flecainide and propafenone [15]. However, these data pertain to short-term class Ic agent administration in a well-controlled environment, under monitoring; consequently, their applicability to long-term treatment is uncertain, yet hypotheses are being generated.

Focusing on the long-term therapy with flecainide, the Flec-SL trial has shown that long-term (but no longer than 6 months) as compared to short-term use of flecainide is more effective after electrical cardioversion with a comparable safety profile. Interestingly, 6% of the study population suffered from CAD and 13.5% from valvulopathies [18].

The role of flecainide in early rhythm control was notably highlighted in the pivotal EAST-AFNET 4 trial, which demonstrated that early initiation of rhythm control therapy—including antiarrhythmic agents such as flecainide—significantly reduced cardiovascular mortality, stroke, and hospitalizations for heart failure or acute coronary syndrome when compared to usual care [19].

Among the 2789 enrolled patients, 689 received either flecainide (>80%) or propafenone. Even though the trial protocol excluded flecainide in patients with left ventricular ejection fraction <40% and significant left ventricular hypertrophy (>15 mm), flecainide was administered in patients with stable CAD or MI (*n* = 41), stable heart failure (*n* = 177) or mild left ventricular hypertrophy (*n* = 26). Furthermore, any prolongation in QRS duration >25% mandated discontinuation of therapy, emphasizing the need for close electrocardiographic monitoring.

Towards this direction, EAST-AFNET 4 was analyzed post hoc, focusing on the safety and efficacy of flecainide and propafenone in the early rhythm control arm of the trial especially in the one third of the population with SHD [20]. Importantly, flecainide and propafenone were more efficacious and safer compared to those not receiving class Ic antiarrhythmic drugs (AADs) [20]. No increased risk was found in subgroups with stable CAD, stable heart failure, or left ventricular hypertrophy on class Ic AADs.

In contrast to guidelines [1], in the European Rythmol/Rytmonorm Atrial Fibrillation Trial (ERAFT), patients with previous MI were included, and no increased mortality or proarrhythmia was observed in the propafenone arm [21].

More recently, analyses obtained from non-randomized cohorts, have shown that flecainide does not exhibit an increased rate of proarrhythmia or HF events in patients with stable or revascularized CAD when compared with the class III AADs treatment [6]. Burnham et al. conducted a large observational study evaluating long-term outcomes in AF patients with stable CAD but without prior MI who were treated with flecainide. Over a median follow-up of 3 years, flecainide use was associated with significantly lower mortality rates (9.1% vs. 19.3%) and major adverse cardiovascular events (22.9% vs. 36.6%) over three years compared to class III AADs (amiodarone or sotalol). When patients with PCI or CABG were considered, flecainide treatment was associated with lower, although not significantly, adverse events rates compared to the class-III AAD group. These results underscore that, in carefully selected patients, flecainide may offer not only safety but also potential clinical advantage over alternatives traditionally considered safer in SHD populations [6].

Ashraf et al. conducted a retrospective study on AF patients with stable CAD and preserved ejection fraction who received flecainide. The results showed no statistically significant difference in all-cause mortality or sudden cardiac death when compared with non-flecainide users, suggesting that in the absence of prior infarction or significant scar, flecainide may be safe [22]. Individuals with prior MI or significant systolic dysfunction were excluded and findings suggested no significant increase in all-cause mortality or sustained ventricular arrhythmias compared to controls. Consequently, flecainide may be safe in selected CAD patients, particularly when modern imaging is used to exclude scar and active ischemia [22].

Kiani et al. conducted a detailed evaluation of the feasibility and safety of flecainide and other class Ic antiarrhythmic agents in patients with varying degrees of coronary artery disease (CAD) and other structural abnormalities, including left ventricular hypertrophy [23]. The safety of using flecainide or propafenone in 3445 patients with varying degrees of CAD was compared to 2216 CAD patients treated with class III agents (sotalol or dofetilide) exhibiting an improved event-free survival of class Ic AADs. However, patients with obstructive CAD receiving class Ic agents had poorer event-free survival than those on class III agents [23]. These results underscore the critical need to distinguish between stable and high-risk SHD when considering antiarrhythmic therapy, rather than applying blanket contraindications derived from outdated high-risk cohorts [23].

Sangpornsuk et al. offered further evidence through a 2025 retrospective analysis of patients with confirmed SHD who were treated with flecainide. No increased incidence of ventricular tachycardia, ventricular fibrillation, or arrhythmic deaths compared to matched controls were reported [5]. Importantly, these patients had undergone imaging to exclude infarction or active ischemia and had preserved ejection fraction—a critical safety determinant.

The FLECA-ED trial (NCT05549752) is an ongoing RCT designed to compare the efficacy and safety of flecainide versus amiodarone for cardioversion of paroxysmal AF in patients with CAD without residual ischemia and a left ventricular ejection fraction >35% [9]. Preliminary results support the superiority of flecainide over amiodarone in both safety and efficacy [8].

Taken together, the above findings challenge the prevailing dogma and suggest that flecainide may be used safely in a broader segment of the SHD population than currently reflected in guidelines, especially with modern diagnostics guiding risk stratification.

#### 1.1.3. Flecainide Use in Premature Ventricular Contractions and Associated Cardiomyopathy

Flecainide is also increasingly used to treat frequent premature ventricular contractions (PVCs), particularly those originating from the outflow tracts in patients with no apparent structural heart disease [24]. According to the 2022 ESC guidelines, flecainide is recommended as a class IIa treatment for symptomatic idiopathic PVCs when catheter ablation is not preferred or is unavailable although this recommendation is based on scarce data [2,24].

PVC-induced cardiomyopathy is a potentially reversible condition where frequent ectopy leads to left ventricular dysfunction [2,25]. Early intervention to suppress PVCs is essential to prevent long-term systolic failure. In this context, flecainide has demonstrated high efficacy, particularly in patients with preserved ejection fraction and non-scar-related PVC origins [2,24]. The UNIFLECA study (NCT06949748), while focused on idiopathic PVCs, provided compelling evidence of flecainide’s safety and effectiveness in such populations [24].

Beyond idiopathic PVCs, emerging data support flecainide use in selected patients with mild structural abnormalities [26,27]. Hyman et al. assessed the efficacy and safety of flecainide in 20 patients suffering from PVC-induced cardiomyopathy post unsuccessful ablations (7 with myocardial delayed enhancement and 6 with implantable or wearable defibrillator) [26]. Interestingly, flecainide related reduction in PVC burden from 36.2% to 10.0% was accompanied by an increase in left ventricular ejection fraction from 37.4% to 49.0%. in the absence of any ventricular arrhythmias or sudden cardiac deaths [26].

Towards the same direction, Raad et al. examined the safety and efficacy of class Ic AADs in patients with nonischemic cardiomyopathy and implantable cardioverter-defibrillators verifying increase in left ventricular ejection fraction biventricular pacing percentage without compromising safety [27].

#### 1.1.4. Flecainide in ARVC

ARVC is an inherited cardiomyopathy characterized by progressive loss of ventricular myocytes, and their replacement with fibrous and fatty tissue, predominantly in the right ventricle. This pathological process results in ventricular dysfunction and a establishes a substrate that favors the development of ventricular arrhythmias and SCD.

The common use of beta-blockers in ARVC is mostly an extrapolation of the beta-blocker’s efficacy in preventing sudden cardiac death in heart failure; moreover, it relies on observations that VA in ARVC is often effort-related and catecholamine-facilitated [4]. In case of symptomatic VA despite beta-blocker treatment, sotalol or amiodarone can be considered [2].

Several retrospective studies of ICD recipients have analyzed the efficacy of amiodarone and sotalol in ARVC patients, affecting even inducibility during programmed ventricular stimulation [28,29,30,31,32]. Ermakov et al. published a case series of 8 ARVC patients with ICD and refractory VA associated with sotalol (*n* = 5) or metoprolol (*n* = 3), that received flecainide with good efficacy [33]. Roland assessed in a retrospective way one hundred consecutive ARVC patients on both flecainide and beta-blockers [34]. Flecainide was discontinued in only 10%, mostly due to lack of efficacy. Most importantly, flecainide was accompanied by a significant reduction In PVCs burden as well as in inducibility during programmed ventricular stimulation [34].

Likewise, most recently, in another retrospective study, Gain et al. reported that flecainide exhibited significantly reduced complex ventricular arrhythmias being well-tolerated in 92% of cases, irrespective of the underlying genotype and the presence of left ventricular involvement [7]. These findings reinforce and extend the use of flecainide as an adjunct to beta-blockers in ARVC patients with recurrent ventricular arrhythmias, particularly when first-line therapies (beta-blockers, sotalol, or amiodarone) are insufficient or not tolerated [7,35] (Table 1).

#### 1.1.5. Flecainide in LV Hypertrophy (LVH)

Left ventricular wall thickness exceeding 15 mm is classified as LVH and currently constitutes a contraindication for flecainide use. Recently, essential insights have been provided into the application of flecainide in these patients. The retrospective study by Sangpornsuk et al., which assessed 336 patients included 47 patients with SHD, with 28% of those (*n* = 14) with LVH. Throughout the one-year follow-up period, no increase in VA or all-cause mortality was observed [5]. Moreover, an EAST-AFNET 4 trial sub-analysis involved 26 patients with LVH > 15 mm within the class Ic antiarrhythmic drug cohort, reporting no indications of increased safety risks [20].

#### 1.1.6. Pro-Arrhythmic Effect of Long-Term Flecainide Use

Despite these encouraging findings, it is essential to remain vigilant about flecainide’s proarrhythmic potential. Observational data and mechanistic studies indicate that flecainide may facilitate reentrant arrhythmias by slowing conduction velocity and increasing the heterogeneity of refractoriness, particularly in regions of myocardial injury or fibrosis, due to its potent sodium channel blockade [13]. It is associated with a dose- and rate-dependent manner of QRS duration prolongation, reflecting slowed interventricular conduction [36,37]. Furthermore, flecainide promotes 1:1 atrial flutter conduction by decelerating atrial conduction, without a concomitant increase in ventricular refractoriness, thereby allowing rapid atrial impulses to conduct directly to the ventricles [38]. Additionally, unmasking of Brugada syndrome may occur in susceptible individuals due to reduced sodium current in the right ventricular outflow tract [39]. Rarely, QT interval prolongation following flecainide administration has been associated with Torsade de Pointes (TdP) and cardiac arrest, likely due to effects on repolarizing potassium currents [40].

## 2. Discussion

There is a plethora of data supporting the efficacy and safety of flecainide in diverse SHD types challenging the prevailing dogma of current guidelines. Among several data regarding the use of flecainide in patients with SHD, EAST-AFNET 4 exhibited that the benefit of early rhythm control extended to patients with early-stage SHD, provided systolic function and ischemic burden were not compromised as well as QRS duration was not widened >25% [20]. These findings show that patients with stable cardiac comorbidities receiving flecainide (and much less often propafenone) therapy did not have more safety events than patients treated with other antiarrhythmics in the EAST-AFNET 4 trial supporting early medical rhythm control in these patients with high efficacy and a low risk of harm. Of note, patients in the EAST-AFNET 4 trial were treated with the highest recommended doses (200 mg flecainide/day, 600 mg propafenone/day), whereas clinical practice tends to prescribe lower doses [20].

**Figure 1:**
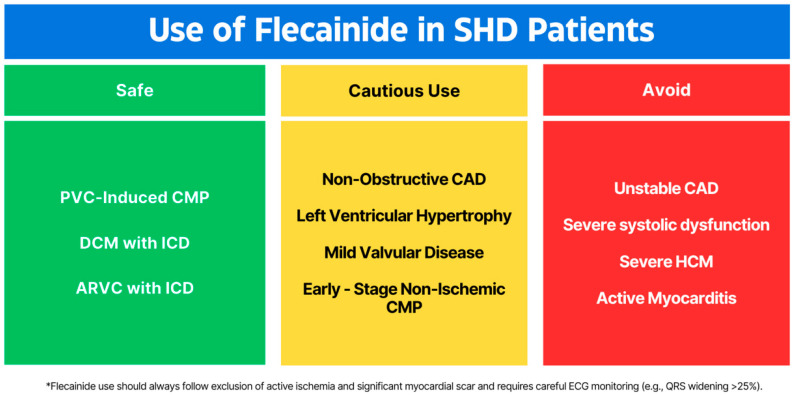
Recommended use of flecainide according to different types of SHD (PVC—premature ventricular contraction, CMP—cardiomyopathy, DCM—dilated cardiomyopathy, ICD—implantable cardioverter defibrillator, ARVC—arrhythmogenic ventricular cardiomyopathy, CAD—coronary artery disease, HCM—hypertrophic cardiomyopathy. A sentence for the Figure 1 is added, the * is used for safety reasons, it is a reminder to use flecainide wisely.

Beyond active ischemia, presence of myocardial scar was also considered a critical factor in triggering lethal arrhythmias in the CAST population [3,10,11,12]. ARVC patients constitute a paradigm of extreme myocardial scar in the absence of ischemia, mostly due to their young age [35]. The common use of beta-blockers in ARVC is mostly an extrapolation of the beta-blocker’s efficacy in preventing sudden cardiac death in heart failure and relies on observations that ventricular arrhythmias in ARVC is often effort-related and catecholamine-facilitated. Superior efficacy of flecainide compared to sotalol in this setting without compromising safety debunks another CAST myth leaving active ischemia as a definite red alarm before its use. A graphical summary of the recommended use of flecainide in different SHD categories, is presented in Figure 1.

### Future Directions

Currently it is suggested that flecainide, when used with appropriate patient selection modern imaging techniques and employment of ECG surveillance, may be underutilized in SHD. However, despite promising observational and registry data, randomized controlled trials (RCTs) remain notably absent in this space. The design and execution of prospective RCTs evaluating flecainide in patients with stable CAD and early-stage cardiomyopathies are urgently needed.

Such trials should employ initially strict inclusion criteria, excluding prior MI, severe systolic dysfunction, or active ischemia, and integrate cardiac magnetic resonance imaging or coronary computed tomography to either confirm structural integrity or demonstrate scar presence below a, currently unknown, threshold. Obviously, ICD bearers with relatively preserved systolic function and thus limited scar presence are a unique population, suitable for assessing flecainide use in the presence of minimal/mild structural disease in as much as they are effectively protected from tachyarrhythmic sudden death. Outcomes should assess both arrhythmic control (AF burden or PVC suppression) and hard safety endpoints such as sudden cardiac death, ventricular arrhythmias, and all-cause mortality.

A pivotal step toward redefining antiarrhythmic management in SHD is the ongoing FLECA-ED trial, which evaluates flecainide head-to-head against amiodarone for pharmacological cardioversion in AF patients with stable CAD and preserved left ventricular ejection fraction [8,9]. The trial’s strict inclusion criteria—excluding prior infarction, active ischemia, or severe systolic dysfunction—directly address one of the most controversial areas of flecainide use. Beyond establishing acute safety, the study may provide definitive evidence supporting the role of flecainide in the emergency management of AF, particularly in patients who are not candidates for immediate electrical cardioversion or catheter ablation. FLECA-ED has the potential to cause future guideline revisions and clinical protocols, expanding use of flecainide beyond the current “no-structural-heart-disease” paradigm and into carefully selected SHD populations [19].

## 3. Conclusions

Although traditionally contraindicated in SHD, flecainide is experiencing a cautious resurgence, supported by recent observational studies, registry data, and precision medicine approaches. Modern imaging techniques now permit rigorous stratification of SHD subtypes, allowing clinicians to distinguish between high-risk and stable structural phenotypes. Evidence from studies in stable CAD, dilated cardiomyopathy, and ARVC consistently indicates that flecainide can be both effective and safe when used under stringent clinical criteria. The growing body of evidence supports a reappraisal of the role of flecainide in SHD. Prospective randomized trials are now imperative to confirm these findings and potentially reshape clinical guidelines concerning antiarrhythmic therapy.

## Figures and Tables

**Table 1:** Summary Table of Flecainide Use in SHD Patients.

SHD Type	Study—Year	Analysis Type	Enrolled	On Flecainide	Patients with SHD	Follow-Up	Mortality/Discontinuation	Arrhythmia-Related Events
Ischemic Heart Disease (CAD/MI)	CAST [3], 1989	Original RCT	2309	730	730—Post-MI with LV dysfunction	Mean 10 months	↑ All-cause mortality (56 arrhythmic deaths)—Non-Q Wave related MI	Excess ventricular arrhythmias
	Tsiachris et al., 2021 [14,15]	Network meta-analysis & systematic review	3310	580	113—Ischemic heart disease	N/A	None	2 VT, 4 bradycardia, 31 hypotension
	Burnham et al., 2022 [6]	Retrospective cohort (AF + CAD)	3445	328	196—Stable CAD.	Median: 3 yrs	Stable CAD: 18 deaths.	Stable CAD: 11 VT.
					134—Post PCI/CABG		Post PCI/CABG: 28 deaths	Post PCI/CABG: 15 VT
	FLECA-ED [8,9], 2023	Prospective RCT (AF cardioversion in ED)	25	10	10—Stable CAD with LVEF >35%	Acute	TBA	TBA
	FLEC-SL [18]	Prospective Randomized (AF Pharmacologic Cardioversion)	635	601	37—CAD	Up to 6 months	1 Event	No excess VT/VF
					86—Valvular Heart Disease			
	EAST-AFNET 4, 2020–24 [20]	RCT subanalysis (early rhythm control)	2789	689 (Class IC)	41—Stable CAD; 177—HFpEF	Median 5 years	Part of 34 composite events (death/stroke/RC-related); no excess in Class IC subgroup	No excess VT/VF
	Ashraf et al., 2022 [22]	Retrospective cohort (AF + CAD)	348	348	196—Obstructive CAD (>70% stenosis or PCI/CABG)	Mean 6.3 years	15 deaths/cardiac arrests	No increase in proarrhythmia overall—VT/VF in 15 patients
					152—Non-obstructive CAD (<50% stenosis)		10 deaths/cardiac arrests	VT/VF in 10 patients
	Kiani et al., 2023 [23]	Multicenter retrospective (Class IC vs. Class III)	5661	3445 (Class IC)	Subgroup: obstructive CAD, LVH	Long-term	Worse survival in obstructive CAD subgroup than Class III AADs	↑ MACEs in High-Risk CAD subgroup
	Sangpornsuk et al., 2025 [5]	Retrospective cohort	336	336	Broad SHD (5—CAD, 13—LVH,12—↓ LVEF, 4—Valvular Heart Disease)	Long-term	2 Non-Cardiac Deaths	No ↑ VT/VF vs. Non SHD Group
PVC-Induced Cardiomyopathy/NICM	Raad et al., 2018 [26]	Retrospective (PVC-CMP)	34	23	34—NICM with ICD	29 Months mean	29% Discontinuation	PVC burden ↓ 20 → 6%; LVEF ↑ 33 → 37%; 2 sustained VT, 1 atrial flutter, AF stable in most
	Hyman et al. [18]	Retrospective (PVC-induced CMP)	20	13	NICM—PVC induced CMP (Mean EF: 37%)	3.8 Years Mean	8/20 discontinued (inefficacy/side effects)	No sustained VA; PVC burden ↓ 36 → 10%; EF ↑ 37 → 49%
Arrhythmogenic Right Ventricular Cardiomyopathy (ARVC)	Gain et al., 2025 [7]	Multicenter retrospective	191	191	191—ARVC (59% ICD)	Median 4.2 years	0 deaths; 8% discontinued	↓ PVC burden; ↓ NSVT; No sustained VA; minor symptoms
	Ermakov, et al. [33]	Retrospective Case series	45	8 with Sotalol/Metoprolol	ARVC	Median 35.5 Months	No deaths reported; discontinuation/AEs not clearly stated	6/8 arrhythmia-free; 2/8 recurrent arrhythmia requiring repeat ablation
	Roland, et al. [34]	Retrospective Cohort	100	100	ARVC	Median 47 Months	No deaths: ~10% discontinued (6 inefficacy, 1 AF, 3 side effects)	↓ PVC burden; ↓ PVS positivity (94% → 40%); sustained VA rate ~5% at 1 yr, ~25% at 5 yr under treatment
Left Ventricular Hypertrophy (LVH)	EAST-AFNET-4 [20]	RCT subanalysis (early rhythm control	2789	689 (Class IC)	26—LVH (>15 mm)	Median 5 years	No excess mortality in LVH subgroup	No excess VT/VF
	Sangpornsuk et al., 2025 [5]	Retrospective cohort	336	336	13—with LVH > 14 mm	1 year	No increase in all-cause mortality	No increase in VA compared to non-SHD group

MI: Myocardial Infarction; AF: Atrial Fibrillation; CAD: Coronary Artery Disease; RCT: Randomized Controlled Trial; LV: Left Ventricle; ↑: Increase/↓: Decrease, NICM: Nonischemic CMP, VT: Ventricular Tachycardia; VF: Ventricular Fibrillation; VA: Ventricular Arrhythmia.

## Data Availability

No original data presented herein. All data were retrieved from studies publicly available.
